# Supplementary material for: Modulation of cannabinoid receptor 2 alters neuroinflammation and reduces formation of alpha-synuclein aggregates in a rat model of nigral synucleinopathy
Source: bioRxiv. 2024 Mar 23:2023.08.25.554814. Preprint. [Version 2] doi: 10.1101/2023.08.25.554814 (PMC10983852; doi:10.1101/2023.08.25.554814)
Supplement: Supplement 1 — Figure S1. Flow gating strategy for rat brain immune cells. Cells were initially gated by SSC and FSC, doublets excluded and live cells selected as those that did not take up live/dead fixable stain. Live cells were further gated by CD45 and CD11b where microglia (CD45lo, CD11b+), lymphocyte (CD45+CD11b−), and monocytes (CD45hi, CD11b+) populations were identified. Microglia subsets were further defined by MHCII, CD172a and CD11b frequencies and MFI. Monocytes were gated on CD3−, and further defined by CD11b+/CD172a+. Two populations of monocytes were identified CD43lo (classical monocytes) and CD43hi (non-classical monocytes) and monocyte activation determined by frequencies and MFI of MHCII and CD172a. Lymphocytes were gated as CD3+ and further defined by CD4 and CD8 expression. Figure S2. Flow gating strategy for rat PBMC myeloid cells. Cells were initially identified by SSC and FSC, doublets excluded and live cells selected as those that did not take up live/dead fixable stain. Immune cells were gated as CD45+ and monocytes further defined by CD3−. Granulocytes were excluded by size with high SSC, and monocytes further defined by CD11b+/CD172a+. Two populations of monocytes were initially identified by CD43 and further defined by His48 expression: classical monocytes (CD43lo/HIS48+) and non-classical monocytes (CD43hi/His48lo) and monocyte activation determined by MHCII MFI. Figure S3. Flow gating strategy for rat lymphocytes and elevation of peripheral Tregs in SMM-189-treated rats (high cohort) after 7 weeks of treatment. Cells were initially identified by SSC and FSC, doublets excluded and live cells selected as those that did not take up live/dead fixable stain. Lymphocytes were gated as CD3+ and subpopulations of T cells defined by CD8 or CD4. Tregs were also identified from CD4 populations (foxp3+ CD4+) with subpopulations separated by CD25 expression. B cells were identified as CD3−CD45RA+ populations. PBMCs were evaluated by flow cytometry at baseline, 4 [file media-1.pdf]

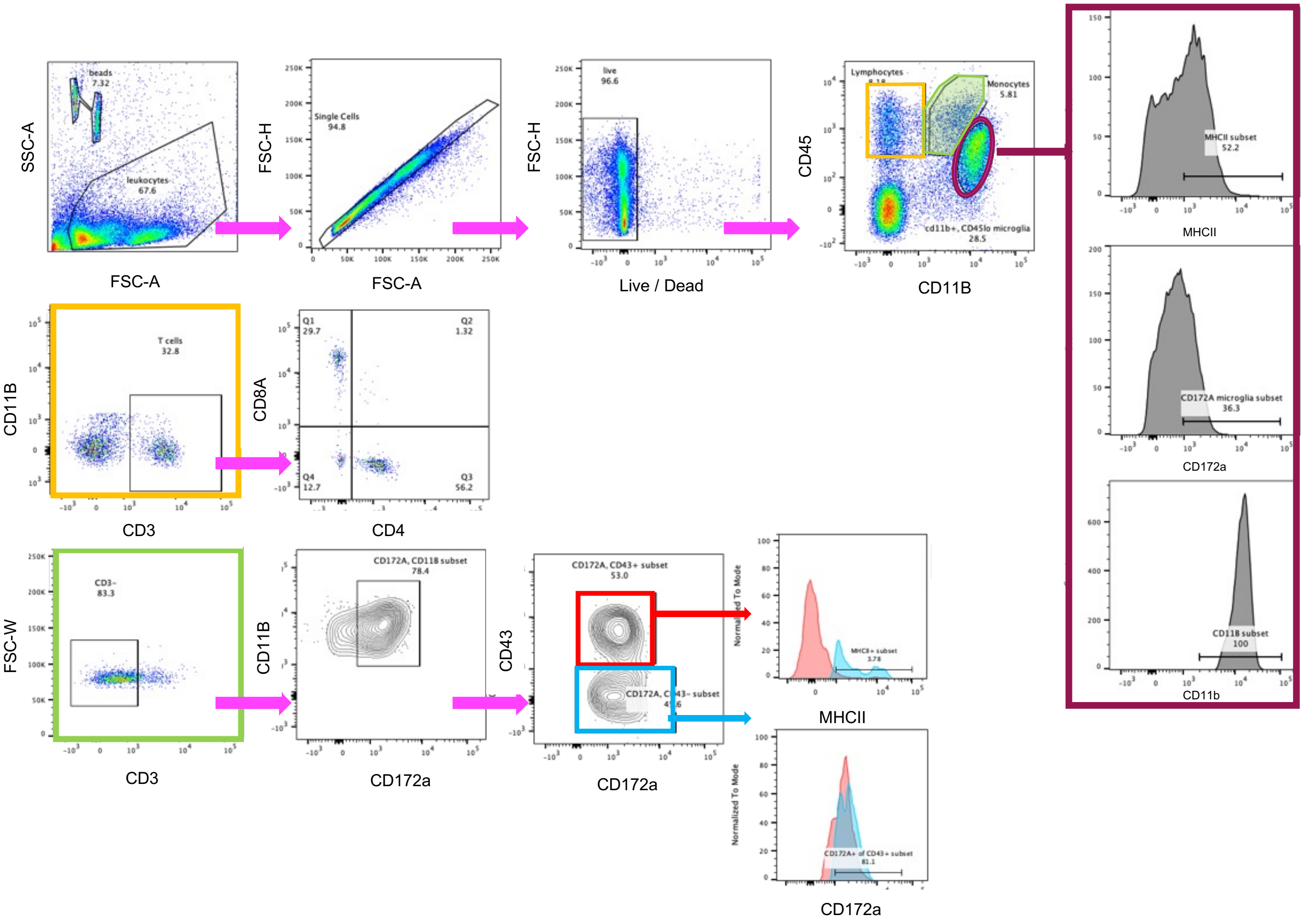

Figure S1. ***Flow gating strategy for rat brain immune cells***

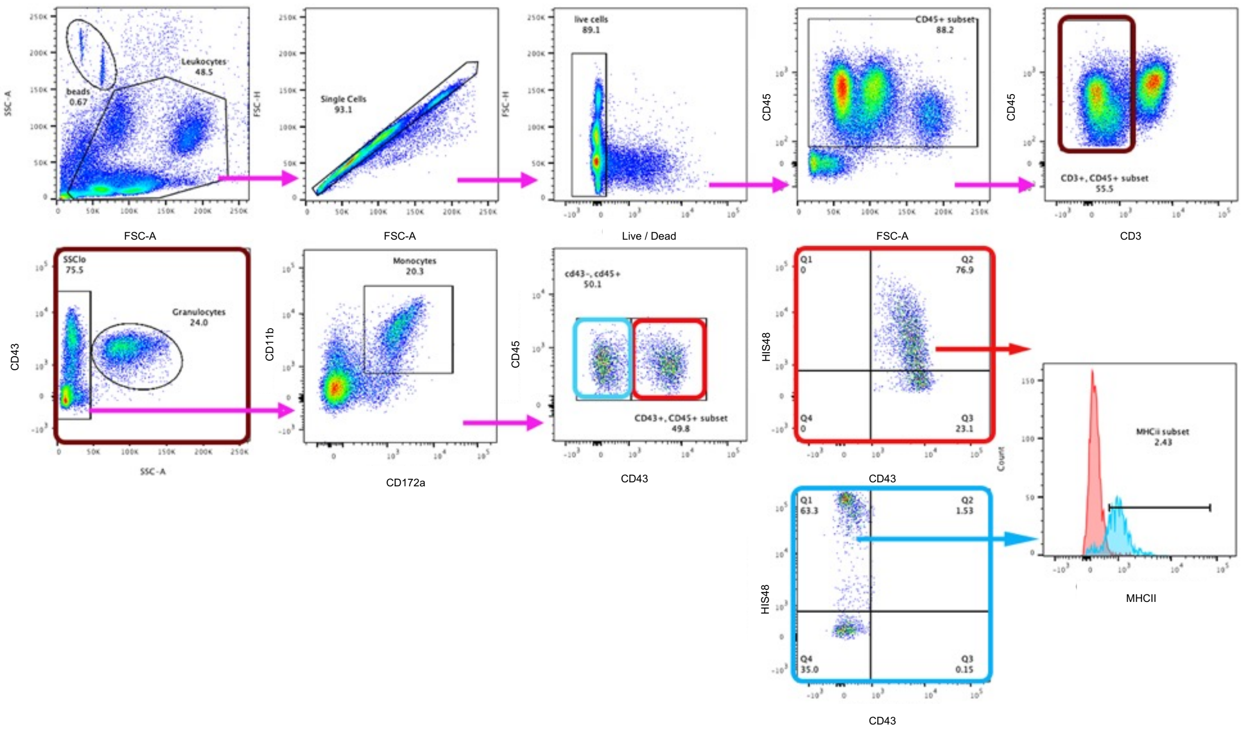

Figure S2. ***Flow gating strategy for rat PBMC myeloid cells***

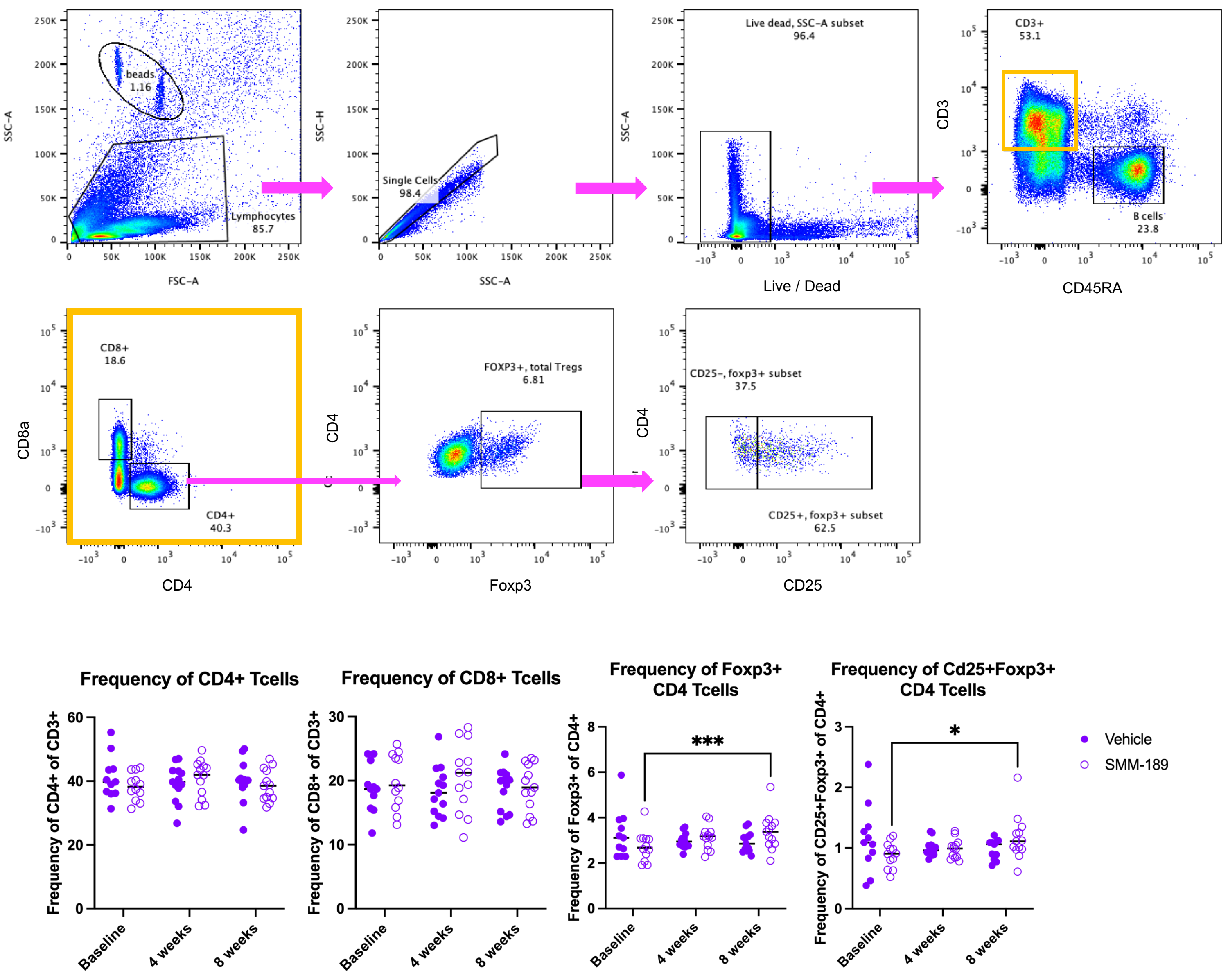

Figure S3. Flow gating strategy for rat lymphocytes and elevation of peripheral Tregs in SMM-189-treated rats (high cohort) after 7 weeks of treatment

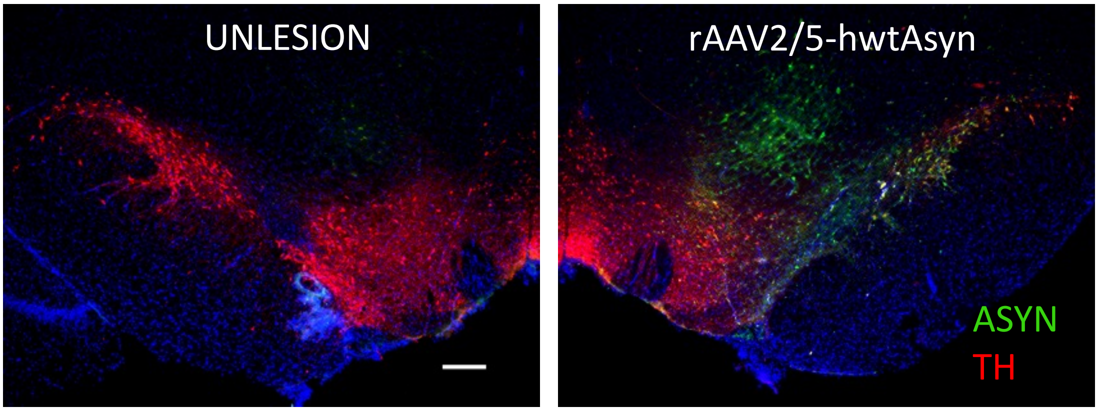

Figure S4. Targeting of rAAV2/5-hwtAsyn in the rat substantia nigra

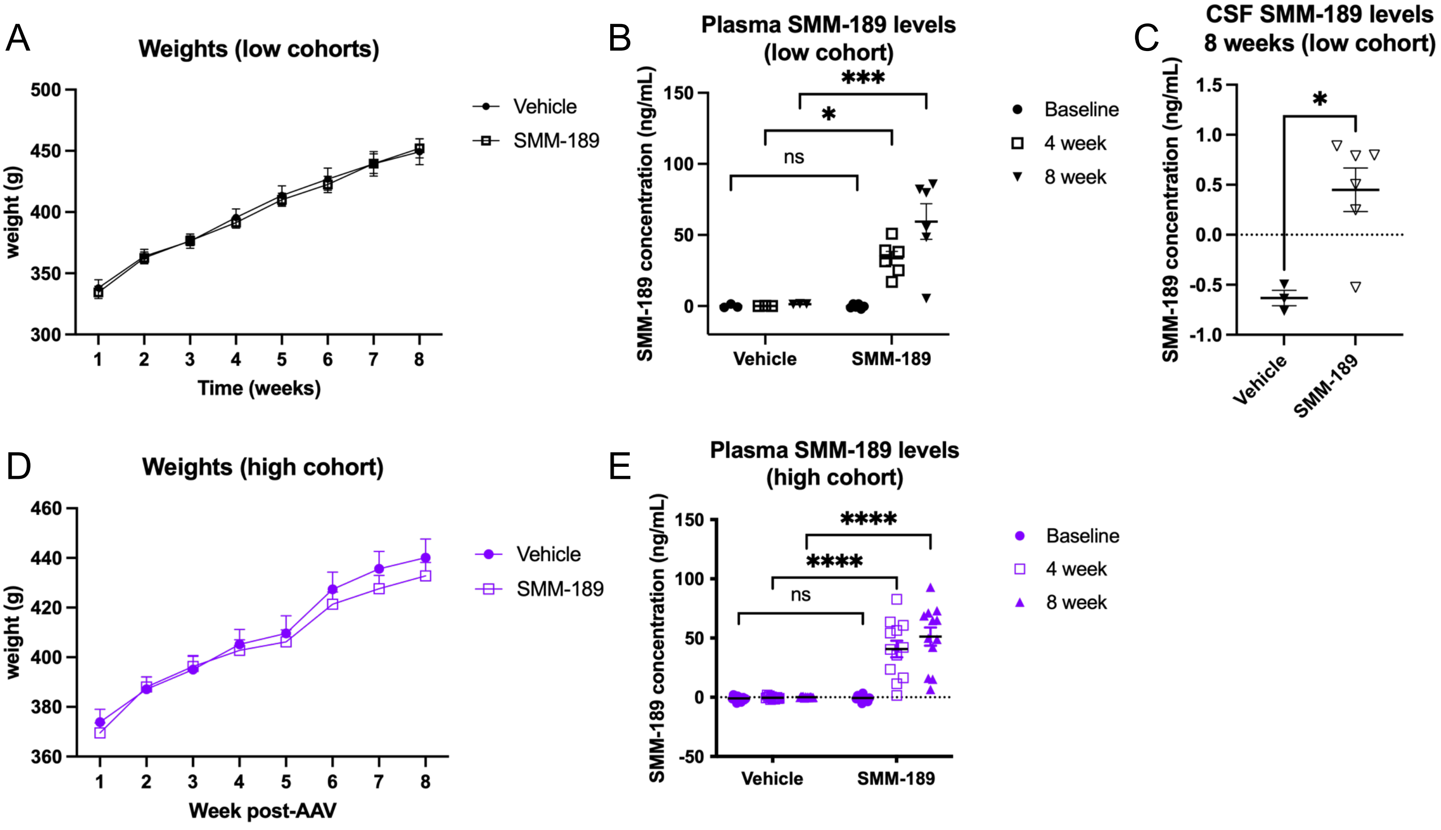

Figure S5. Animal body weights and levels of SMM-189 in plasma and CSF

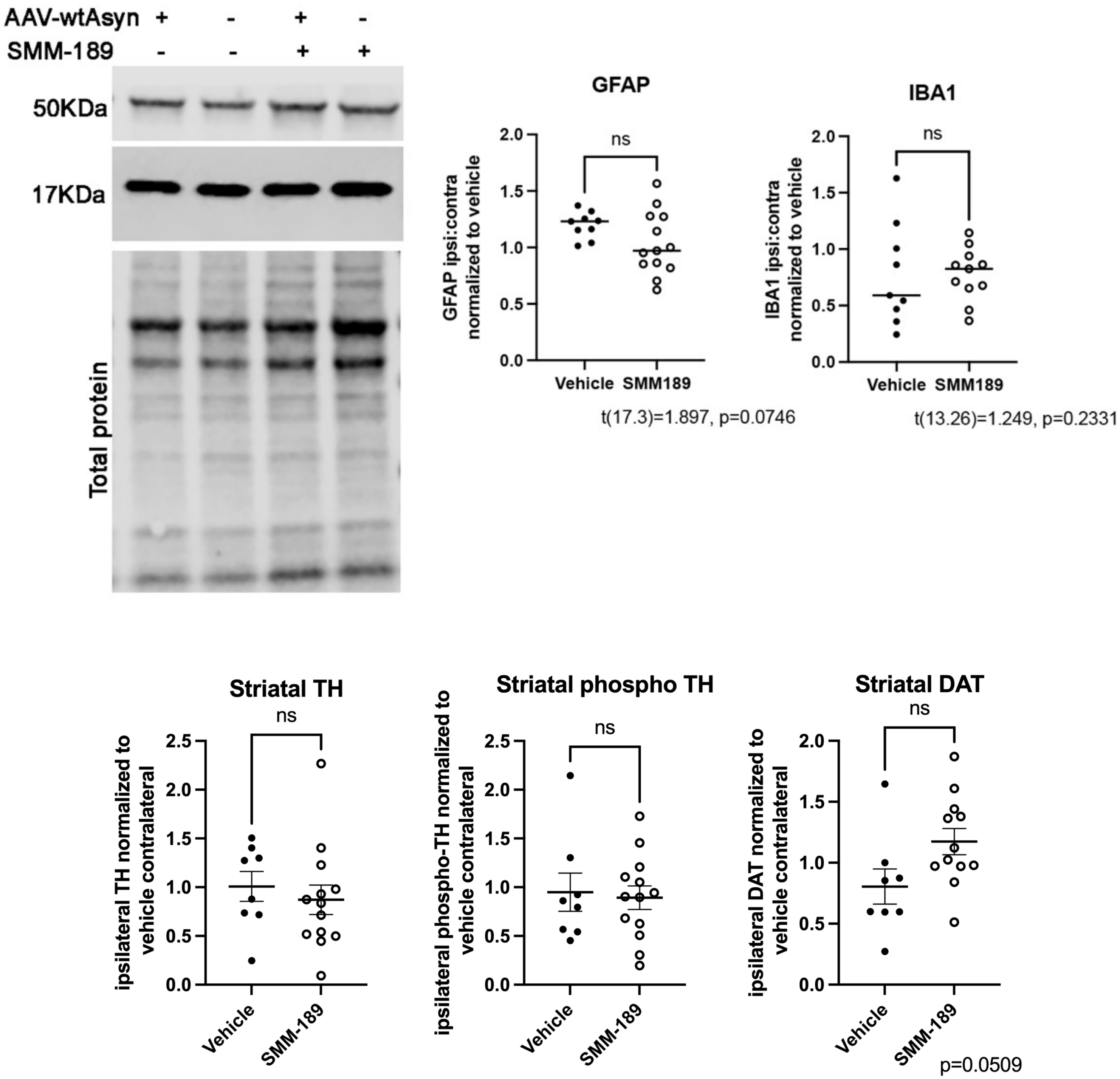

Figure S6. Protein markers for astrocytes, microglia or dopaminergic neurons in striatum do not change by CB2 modulation with SMM-189

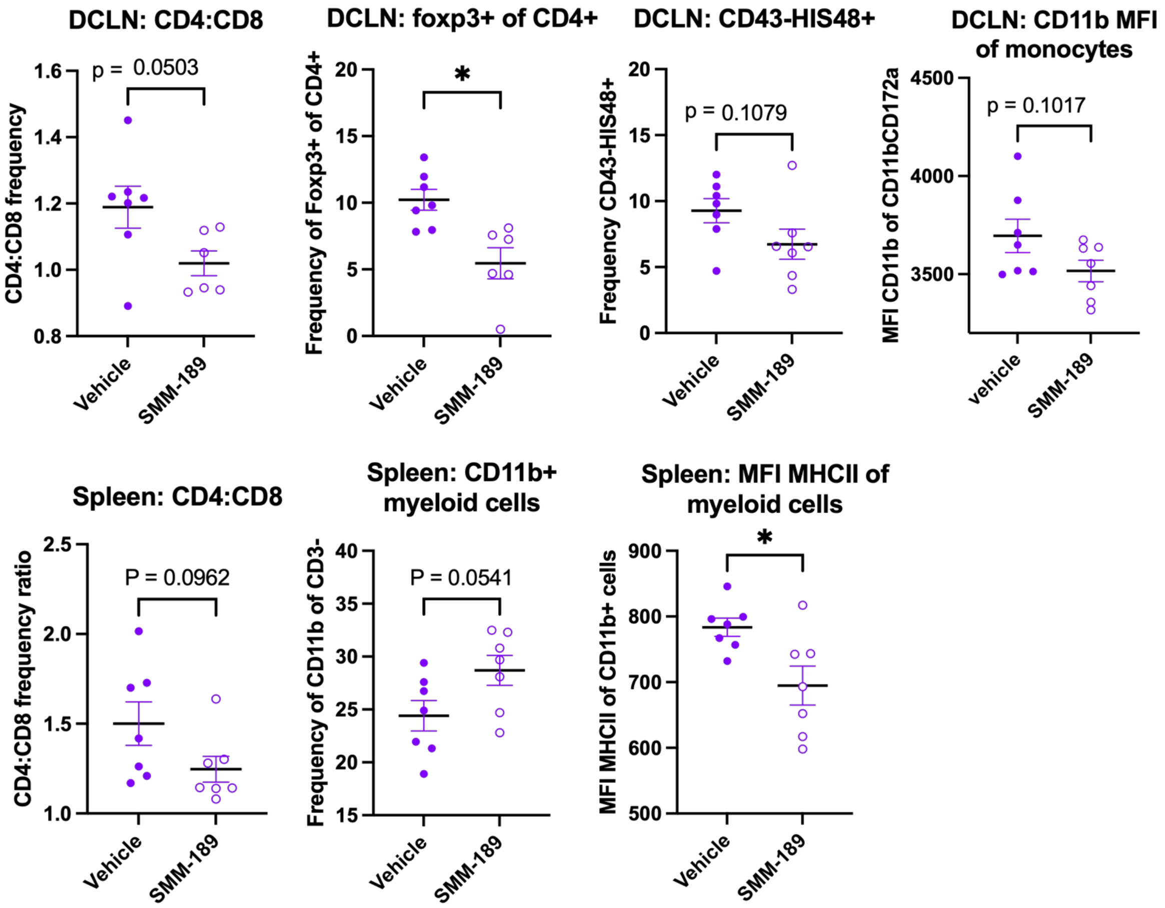

Figure S7. Immune cell populations in deep-cervical lymph nodes (DCLN) and splenocytes are minimally affected in SMM-189-treated rats (high cohort) after 7 weeks of dosing
