# Supplementary material for: Modulation of cannabinoid receptor 2 alters neuroinflammation and reduces formation of alpha-synuclein aggregates in a rat model of nigral synucleinopathy
Source: bioRxiv. 2024 Mar 23:2023.08.25.554814. Preprint. [Version 2] doi: 10.1101/2023.08.25.554814 (PMC10983852; doi:10.1101/2023.08.25.554814)
Supplement: Supplement 2 [file NIHPP2023.08.25.554814v2-supplement-2.pdf]

bioRxiv preprint doi: https://doi.org/10.1101/2023.08.25.554814; this version posted March 23, 2024. The copyright holder for this preprint (which was not certified by peer review) is the author/funder, who has granted bioRxiv a license to display the preprint in perpetuity. It is made available under aCC-BY 4.0 International license.

Table S1. Myeloid PBMC flow panel

Antibody	Manufacturer	Product number	Dilution
anti-Rat CD45-BUV395	BD Biosciences	740258	1:50
anti-Rat His48-FITC	Invitrogen	11-0570-82	1:200
Anti-Rat CD43-PE-Vio770	Miltenyi	130-107-721	1:100
Anti-rat CD3-Viogreen	Miltenyi	130-103-126	1:200
Anti-rat CD4-APC-Vio770	Miltenyi	130-107-504	1:100
Anti-ms/rat MHCII-PerCP-Vio700	Miltenyi	130-107-877	1:100
Anti-rat CD11b-Pac Blue	BioRad	MCA275PB	1:50
Anti-rat RP1-BV786	BD Biosciences	743058	1:20
Anti-rat CD172a-APC	R&D systems	FAB7307A	1:100
Anti-rat CD32	BD Biosciences	550271	1:100

Table S2. Lymphoid PBMC flow panel

Antibody	Manufacturer	Product number	Dilution
Anti-rat CD45RA-PECy7	Biolegend	202315	1:200
Anti-rat CD3-Viogreen	Miltenyi	130-103-126	1:200
Anti-rat CD4-APC-Vio770	Miltenyi	130-107-504	1:100
Anti-CD8a-PerCP-Vio700	Miltenyi	130-108-914	1:100
Anti-rat CD25-BV786	BD Biosciences	742757	1:50

bioRxiv preprint doi: https://doi.org/10.1101/2023.08.25.554814; this version posted March 23, 2024. The copyright holder for this preprint (which was not certified by peer review) is the author/funder, who has granted bioRxiv a license to display the preprint in perpetuity. It is made available under aCC-BY 4.0 International license.

Anti-ms/rat Foxp3-AF647 *stained following intracellular permeabilization	R&D systems	FAB7307A	1:50
Anti-rat CD32	BD Biosciences	550271	1:100

Table S3. Brain Immune cell flow panel

Antibody	Manufacturer	Product number	Dilution
anti-Rat CD44H-FITC	Miltenyi	130-107-854	1:50
Anti-Rat CD62L-PE	BD Biosciences	551398	1:200
Anti-rat CD45RA-PECy7	Biolegend	202315	1:200
Anti-rat CD3-Viogreen	Miltenyi	130-103-126	1:200
Anti-rat CD4-APC-Vio770	Miltenyi	130-107-504	1:100
Anti-CD8a-PerCP-Vio700	Miltenyi	130-108-914	1:100
Anti-rat CD25-BV786	BD Biosciences	742757	1:50
Anti-ms/rat Foxp3-AF647 *stained following intracellular permeabilization	R&D systems	FAB7307A	1:50
Anti-rat CD32	BD Biosciences	550271	1:100

Table S4. Western and IHC antibodies

**Antibody**	**Company**	**Product #**	**Loading protein concentration**	**Concentration**
**Western antibodies**				
TH	Millipore	AB152	10ug	1:2000
Asyn (4B12)	BioLegend	807801	10ug	1:500
DAT	Novus Bio	NBP2-22164	10ug	1:2000
IBA1	Abcam	AB5076	10ug	1:1500
Phospho-TH	Phospho solutions	P1580-40	10ug	1:1000
NFκB p65	Santa Cruz	SC327	15ug	1:500
GFAP	Dako	Z0334	15ug	1:2500
pSer129 (EP1536Y)	Abcam	AB51253	15ug	1:500
**IHC antibodies**				
IBA1	Wako	019-19741		1:500
TH	Immunostar	22941		1:1000
Asyn (4B12)	BioLegend	807801		1:2500
pSer129	Abcam	AB51253		1:2500

bioRxiv preprint doi: https://doi.org/10.1101/2023.08.25.554814; this version posted March 23, 2024. The copyright holder for this preprint (which was not certified by peer review) is the author/funder, who has granted bioRxiv a license to display the preprint in perpetuity. It is made available under aCC-BY 4.0 International license.

(EP1536Y)				
CD68	Sertec	MCA341R		1:2000
MHCII	Biorad	MCA46GA		1:1000
CD163	Biorad	MCA342R		1:1000
